# Exogenous Ferulic Acid Mitigates Flooding Stress in Broccoli via GSH-Mediated Redox Homeostasis

**DOI:** 10.3390/plants15091323

**Published:** 2026-04-25

**Authors:** Marta Frlin, Ivana Šola

**Affiliations:** Department of Biology, Faculty of Science, University of Zagreb, 10000 Zagreb, Croatia; marta.frlin@biol.pmf.unizg.hr

**Keywords:** flavonols, glutathione, hydrogen peroxide, kaempferol, metabolic priming, proanthocyanidins, sinapic acid, soluble sugars, water stress

## Abstract

Climate change is increasing flood frequency, exposing plants to severe stress. This study investigated the biostimulant-like effects of exogenous ferulic acid (FA; 1, 10, and 100 mg/L) on broccoli (*Brassica oleracea* var. *cymosa*) microgreens under regularly watered (RW) and flooded (F) conditions. Spectrophotometric, HPLC, and statistical analyses showed that all FA concentrations increased total phenolics and proanthocyanidins in flooded plants, while only 100 mg/L increased proanthocyanidins in RW plants. FA at 1 and 100 mg/L reduced soluble sugars in RW broccoli (18% reduction by both FA concentrations) and enhanced antioxidant capacity (measured by ferric reducing antioxidant power assay, FRAP) in flooded plants (8% and 11%, respectively). Only 10 mg/L FA lowered hydrogen peroxide in RW plants. Flooding significantly decreased glutathione (GSH) levels, but FA treatment doubled GSH concentration and restored its level in flooded broccoli, improving redox balance. FA also influenced individual polyphenols more strongly in RW plants, with notable increases in sinapic acid and kaempferol. Overall, FA enhanced antioxidant status and redox homeostasis under flooding stress, mainly by stimulating glutathione accumulation and phenylpropanoid metabolism. Its regulatory effects were strongly dependent on soil water conditions. These findings underscore the practical and agronomic potential of FA as an effective approach to enhance crop resilience under climate change.

## 1. Introduction

Flooding, as a type of abiotic stress, can have severe consequences on plant growth, development and yield. It results in partial or complete submergence stress and induces oxygen-deficient conditions, which alter plant metabolism [1]. Excess soil moisture and submergence limit gas exchange between the plant and its environment due to the low diffusion rates through the floodwater [2]. Consequently, changes in gene transcripts, proteins, and metabolism occur rapidly. The shift from aerobic to anaerobic metabolism leads to energy exhaustion and is responsible for the impairment of plant development [3]. The decline in root metabolism limits water transport, leading to lower transpiration and photosynthesis rate [2]. Although flooding is a natural occurrence, anthropogenic effect and climate change are expected to increase flood risk [4] leading to more frequent and severe plants stresses. Plants have developed multiple anatomical, morphological, physiological and biochemical mechanisms to cope with flooding stress. Physiological responses include the closing of stomata, reduction in transpiration and inhibition of photosynthesis [5]. Flooding stress leads to the accumulation of reactive oxygen species (ROS) and oxidative stress. In response, plants activate both enzymatic (e.g., catalase, glutathione reductase, peroxidase) and non-enzymatic antioxidants (e.g., proline, glutathione, ascorbic acid) antioxidant systems [6]. An important part of the stress response is the accumulation of polyphenolic compounds, which act as ROS scavengers [7]. Numerous natural plant polyphenolics, acting as endogenous signaling molecules involved in the induction of biotic and abiotic stress tolerance, have also been applied exogenously to enhance plant tolerance to stress [7].

Ferulic acid (FA) is a hydroxycinnamic acid naturally found in many plant tissues. It has potent antioxidant and free-radical scavenging properties and helps protect cells from ROS damage [8]. Its biosynthesis in plants is developmentally regulated; for example, in broccoli, the highest concentration of FA was recorded at the seedling stage [9], whereas in Chinese cabbage it is highest in plants containing six developed leaves [10]. FA is also an important structural component in cell wall that enhances its rigidity and strength by cross-linking polysaccharides and lignin, and protects against microbial degradation [11]. Plants synthesize FA in the phenylpropanoid pathway, and this biosynthetic pathway is stimulated by various environmental stresses [12]. It has been shown that FA is accumulated under drought [13], flooding [14], high environmental temperature [9,15,16], heavy metal [17] and methyl jasmonate stress [18]. Some biotic stresses, such as pathogen invasion and herbivory, also promote FA synthesis [19,20,21]. Apart from its synthesis, FA can also be released from cell walls by the action of enzymes such as esterases and peroxidases, which also contributes to its accumulation in stress conditions [22].

FA operates through multiple interconnected mechanisms to alleviate stress damage and enhance plant tolerance. It acts as a powerful antioxidant and scavenges ROS generated during stress conditions. FA antioxidant activity is a result of its structure (related to the availability of hydroxyl groups) and ability to form stable phenoxyl radicals in reaction with radical molecules [23]. The phenolic ring, characterized by high resonance stability, allows it to receive electrons from free radicals more easily [24]. Importantly, it can also inhibit enzymes involved in free-radical generation and enhance the activity of other scavenging enzymes [25]. Another mechanism of action is the chelation of metallic ions [26], which helps prevent these metals from catalyzing further ROS generation and is especially important in heavy metal stress. It has been suggested that FA can act as hydrogen donor and protect cell membranes from the auto-oxidation process. Additionally, FA can also regulate the expression of genes involved in the stress response [27].

Several studies have evaluated the biostimulant-like potential of FA in mitigating abiotic stress. Application of FA has been shown to improve tolerance to elevated temperatures in maize (*Zea mays* L.) and blueberry (*Vaccinium corymbosum* L.) [28,29]. Exogenous FA enhanced cold resistance in tomato (*Solanum lycopersicum* L.) by upregulating the expression of key genes involved in stress-responsive transcriptional pathways [27]. It also conferred tolerance against excess boron in wheat (*Triticum aestivum* L.) leaves [30], and foliar application of FA improved cadmium detoxification and reduced its accumulation in wheat [31]. Exogenous application of several phenolic acids, including FA, has been shown to mitigate salinity-induced oxidative damage in wheat seedlings [32], while foliar application of FA alleviated salt stress in Chinese cabbage (*Brassica rapa* L. ssp. *pekinensis* (Lour.) Hanelt cv. Cantonner Witkrop) [33]. Furthermore, FA pretreatment enhanced dehydration-stress tolerance in cucumber (*Cucumis sativus* L.) seedlings [34]. FA has been identified as a pivotal metabolite in drought stress response in endemic epiphytic orchid *Dendrobium sinense* and shown to enhance drought survival in *Arabidopsis thaliana* [35]. In addition to abiotic stress, exogenous FA can also help plants cope with biotic stress. It has been shown to offer protection against plant pathogen *Erysiphe pisi* in pea (*Pisum sativum* L.) [36]. Most of these effects are associated with the regulation of oxidative stress through the induction of both enzymatic and non-enzymatic antioxidant systems, leading to reduced hydrogen peroxide levels. Enhanced accumulation of phenolic compounds is also frequently observed.

The results from our previous study showed that concentration of FA was significantly elevated in broccoli microgreens subjected to flooding, which suggests that it could have a key role in mediating stress tolerance in these conditions [14]. Therefore, the aim of this study was to evaluate the potential of exogenous FA to modulate the defense responses and flood tolerance of broccoli (*Brassica oleracea* L. convar. *botrytis* (L.) Alef. var. *cymosa* Duch.) and assess its role in stress response. For that purpose, we analyzed the effects of different concentrations of exogenous FA (1, 10, 100 mg/L) under regular watering and flooding on different groups of polyphenolic compounds, soluble sugars, antioxidant potential, H_2_O_2_, glutathione, and selected individual polyphenolic compounds (caffeic acid, ferulic acid, sinapic acid, quercetin and kaempferol).

## 2. Results and Discussion

### 2.1. Effect of Exogenous Ferulic Acid on Phenolic Content in Broccoli Microgreens Grown Under Regular Watering and Flooding

Exogenous FA affected the polyphenolics profile of broccoli microgreens differentially under regular watering regime and flooding (Table 1). FA did not change the concentration of total phenolics in regularly irrigated plants. On the other hand, it increased them in plants subjected to flooding, and the increase was in correlation with higher concentration of FA applied. Additionally, plants treated with 10 mg/mL FA had significantly higher level of total phenolics when subjected to flooding than under regular watering. Under regular watering, when microgreens were not exposed to stress, exogenous FA did not change the content of total phenolics, whereas under flooding stress FA led to a significant increase in phenolics. Similar results were recorded in maize under heat stress and in Chinese cabbage under salt stress; exogenous FA application resulted in higher levels of total phenolics leading to higher stress tolerance [28,33]. The accumulation of phenolics under various stress conditions is expected, given their multiple important roles in conferring stress tolerance.

Application of 1 mg/L and 100 mg/L FA resulted in notably higher concentration of total phenolic acids in regularly irrigated plants. However, this effect was not observed in plants subjected to flooding. Flooded broccoli microgreens treated with 100 mg/L FA had lower levels of total phenolic acids compared to regularly watered plants. Total hydroxycinnamic acids amount was not significantly affected by exogenous application of FA neither under regular watering nor under flooding. However, broccoli microgreens treated with 1 mg/L and 10 mg/L FA had significantly more hydroxycinnamic acids under flooding than regular watering conditions. A tendency of increase was observed in control plants and those treated with 100 mg/L FA. For comparison, in pea plants foliar application of FA led to an increased activity of phenylalanine ammonia lyase, a key enzyme in phenolics biosynthetic pathway, and higher phenolic acids content, which resulted in improved pathogen resistance [36]. Higher content of total phenolic and hydroxycinnamic acids was also involved in exogenous FA-mediated improvement of salt stress tolerance in Chinese cabbage [33].

Exogenous FA did not significantly affect the concentration of total flavonols neither under regular watering nor under flooding, but control plants and those treated with 1 mg/L and 10 mg/L FA subjected to flooding had significantly higher levels compared to regularly watered plants. For comparison, when broccoli sprouts were subjected to flooding for 7 days, the amount of total flavonols decreased [14]. In Chinese cabbage plants, salt stress significantly reduced the amount of total flavonols, but foliar application of FA elevated them to an even higher level [33]. Total tannins were not significantly affected by any treatment. On the other hand, total proanthocyanidins were affected by both FA and flooding. Under regular watering regime, only highest concentration of FA (100 mg/L) notably increased the concentration of proanthocyanidins. Under flooding conditions, treatment with FA resulted in an increase in proanthocyanidins content at all concentrations tested. However, this increase was not concentration-dependent, as the highest increase was observed at 1 mg/L, whereas the lowest was recorded at 10 mg/L. Interestingly, flooded control plants and those treated with 10 mg/L and 100 mg/L FA had lower levels of total proanthocyanidins than regularly watered plants. Similarly, total proanthocyanidins were also reduced in 7 days flooded broccoli sprouts [14]. Proanthocyanidins are potent antioxidants and free-radical scavengers [37], and as such they can be important for minimizing oxidative damage related to different stress conditions. When broccoli sprouts were subjected to flooding for 7 days, it significantly decreased the amount of all groups of phenolics [14]. However, in the present study most of the polyphenolic groups showed a tendency of increase and flavonols were significantly increased, indicating that the duration of flooding stress is an important factor determining the level of phenolic compounds.

### 2.2. Effect of Exogenous Ferulic Acid on Soluble Sugars in Broccoli Microgreens Grown Under Regular Watering and Flooding

**Figure 1:**
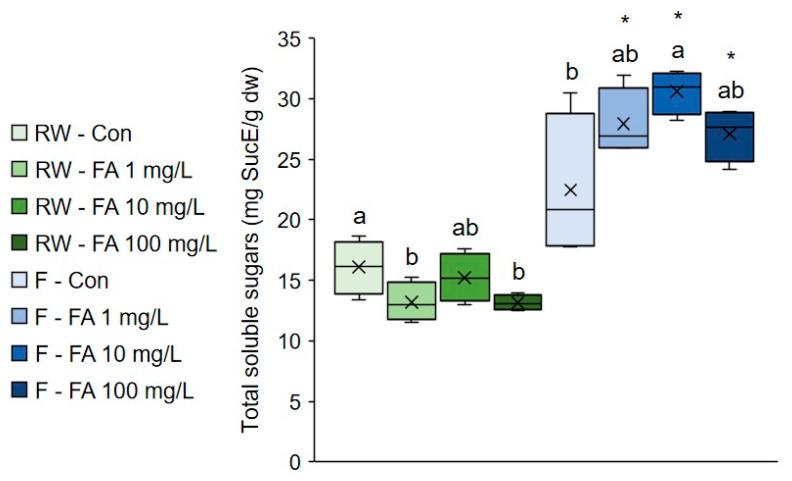
Effect of exogenous ferulic acid on total soluble sugars in broccoli microgreens grown under regular watering and flooding. Values represent mean ± standard deviation of four biological replicates. Different letters indicate a significant difference among plants grown under the same watering regime (one-way ANOVA, Duncan’s test, *p* ≤ 0.05). An asterisk (*) indicates a significant difference between the same treatment group grown under regular watering regime and flooding (Student’s *t*-test, *p* ≤ 0.05). RW = regular watering; Con = control plants irrigated with water; F = flooding; FA = ferulic acid; SucE = sucrose equivalent; dw = dry weight.

Application of 1 mg/mL and 100 mg/L FA significantly decreased the amount of soluble sugars in regularly watered plants, whereas 10 mg/L solution significantly increased their amount under flooding (Figure 1). Concentration of soluble sugars was markedly higher in plants treated with FA under flooding than in regularly watered plants. Soluble sugars are an important component of various stress responses, as they function as osmoprotectants and signaling molecules, and protect cells from oxidative damage by helping maintain cellular redox balance and ROS detoxification [38]. It was proposed that lower consumption and faster accumulation of soluble sugars are mechanisms associated with flooding tolerance [39]. Higher concentration of soluble sugars was observed in flooded plants, even though the increase was not statistically significant in control plants. The higher soluble sugars content in flooded plants treated with FA, relative to control and corresponding regularly watered plants, suggests that exogenous FA results in better flooding tolerance. The accumulation of soluble sugars has also been observed following the exogenous FA application and is associated with improved tolerance to dehydration stress in cucumbers and heat stress in blueberry and maize [28,29,34]. This suggests that soluble sugar accumulation in response to exogenous FA is a general mechanism enhancing tolerance to multiple types of stress and in multiple plant species.

### 2.3. Effect of Exogenous Ferulic Acid on Antioxidant Potential of Broccoli Microgreens Grown Under Regular Watering and Flooding

Antioxidant assays showed different results for the effect of FA on broccoli microgreens under regular watering regime and flooding conditions (Table 2). ABTS (2,2′-azino-bis(3-ethylbenzothiazoline-6-sulfonic acid)) assay revealed no effect of both treatments. On the other hand, the DPPH (2,2-diphenyl-1-picrylhydrazyl) assay showed that microgreens treated with each concentration of FA had lower antioxidant potential than control plants under regular watering conditions, while FA had no effect under flooding. However, all treated plants had significantly higher antioxidant potential under flooding than under normal watering regime. Flooding led to the accumulation of antioxidant molecules in FA-treated microgreens, including different groups of phenolic compounds (Table 1), which formed an important part of the stress response and contributed to the observed increase in antioxidant potential measured by DPPH. On the contrary, FRAP (ferric reducing antioxidant power) assay indicated that FA had no effect in regularly watered plants, whereas 1 mg/L and 100 mg/L FA notably increased antioxidant potential of broccoli microgreens under flooding. Higher antioxidant potential could be attributed to accumulation of total phenolics and proanthocyanidins in response to exogenous FA under flooding (Table 1). Foliar application of FA in Chinese cabbage resulted in higher antioxidant potential measured by DPPH and FRAP under salt stress [33]. Similarly, exogenous FA increased hydroxyl radical scavenging activity of wheat under salt stress [32].

### 2.4. Effects of Exogenous Ferulic Acid on Markers of Oxidative Stress in Broccoli Microgreens Grown Under Regular Watering and Flooding

**Figure 2:**
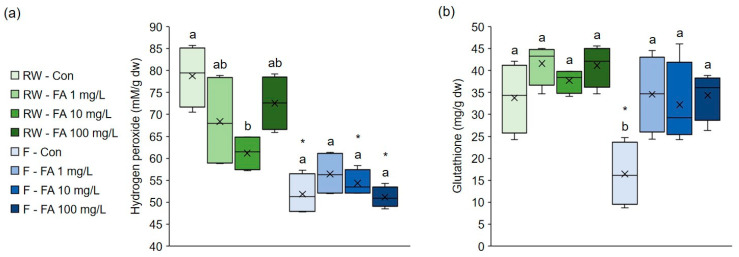
Effects of exogenous ferulic acid on levels of (**a**) hydrogen peroxide and (**b**) glutathione in broccoli microgreens grown under regular watering and flooding. Values represent mean ± standard deviation of four biological replicates. Different letters indicate a significant difference among plants grown under the same watering regime (one-way ANOVA, Duncan’s test, *p* ≤ 0.05). An asterisk (*) indicates a significant difference between the same treatment group grown under regular watering regime and flooding (Student’s *t*-test, *p* ≤ 0.05). RW = regular watering; Con = control plants irrigated with water; F = flooding; FA = ferulic acid; dw = dry weight.

Effects of exogenous FA on markers of oxidative stress, H_2_O_2_ and glutathione (GSH), in broccoli grown under different watering regimes are shown in Figure 2. H_2_O_2_ level was notably lower (−22%) in broccoli microgreens treated with 10 mg/L FA under regular watering (Figure 2a). However, the application of FA did not affect it under flooding. Control plants and those treated with 10 mg/L and 100 mg/L FA had significantly lower levels of H_2_O_2_ under flooding compared to regularly watered plants (−34%, −11% and −30%, respectively). H_2_O_2_, as the most stable ROS, acts as an important signaling molecule in the regulation of plant growth, development and stress responses. However, when overaccumulated, it can cause damage to cellular components. Its accumulation is associated with almost all environmental stresses as part of oxidative stress [40]. Flooding stress increased the H_2_O_2_ level in cauliflower seedlings [41]. In broccoli plants the level of H_2_O_2_ was elevated after 12 h of flooding, but when flooding stress lasted longer than 24 h H_2_O_2_ level was reduced [42]. In our study, flooding resulted in a decrease in H_2_O_2_ content. Flooding results in a lower oxygen concentration in the root zone due to water saturation, limiting aerobic metabolism in which H_2_O_2_ is generated. Thus, reduced oxygen availability under flooding conditions limits the production of H_2_O_2_. We have already observed the reduction in H_2_O_2_ in broccoli sprouts exposed to flooding stress for 7 days [14]. Under flooding conditions, FA did not have a significant impact on H_2_O_2_ level. However, the positive effect of exogenous FA on H_2_O_2_ level, indicating lower oxidative stress, was observed under regular watering regime. Exogenous FA has been shown to reduce H_2_O_2_ level in cucumber under dehydration stress, and in wheat under salt and boron stress [30,32,34].

The GSH concentration was not affected by exogenous FA under regular watering regime (Figure 2b). On the contrary, under flooding, plants treated with all FA concentrations had markedly higher concentration of GSH compared to control plants irrigated with water. Additionally, control plants had significantly lower levels of GSH under flooding compared with regularly watered plants. Obviously, flooding significantly decreased the concentration of the antioxidant GSH, but treatment with FA contributed to its increase and restored the plant’s redox balance to normal levels, even in flood conditions. These findings suggest that the protective effect of exogenous FA in flooding-stressed broccoli is mediated through GSH. The relevance of GSH in protection of plants against abiotic stresses is described in detail in review by Hasanuzzaman et al. [43]. It is emphasized that it increases plant tolerance to drought, salinity, high and low temperature stress, and toxic metals by functioning as non-enzymatic antioxidant, methylglyoxal detoxificator and signaling molecule. Moreover, the potential of GSH exogenous application for improvement of plant properties was tested. For example, Jung et al. [44] showed that exogenously applied GSH can mitigate Cd phytotoxicity in *B. napus*. Ahmad et al. [45] revealed that GSH can be used as a strategy to increase broccoli tolerance to Pb by enhancing nutrient uptake, growth and proline level. However, a practical challenge of such application are high costs of GSH, low stability of GSH in extracellular space and rapid degradation by γ-glutamyl peptidase 1 [46]. On the contrary, FA is much cheaper than GSH and more chemically stable, which suggests it could provide longer-lasting protective effects. This encourages further research into the potential application of FA in protecting plants from water stress. Additionally, it was shown that exogenous FA increased glutathione reductase activity and GSH level, which helped alleviate heat stress in maize and blueberry seedlings, salt and excess boron stress in wheat and dehydration-stress in cucumber seedlings [28,29,30,32,34]. This suggests that exogenous FA-induced GSH accumulation plays an important role in mitigating various abiotic stresses in different plant species. However, GSH functions as a part of a broader network, namely ascorbate/glutathione cycle to preserve redox state by preventing ROS accumulation. The ascorbate/glutathione cycle includes ascorbate, glutathione and NAD(P)H, as well as enzymes ascorbate peroxidase, monodehydroascorbate reductase, dehydroascorbate reductase and glutathione reductase, which function together to detoxify H_2_O_2_ and regenerate reduced ascorbate and glutathione [47]. It is important to emphasize that we analyzed GSH concentration only, whereas other components of the ascorbate/glutathione cycle were not investigated. Therefore, the current study does not provide a complete picture of the ascorbate/glutathione cycle and conclusions on a GSH-mediated mechanism should be interpreted with caution. To better understand the underlying redox mechanisms, more research involving these components is required.

### 2.5. Effects of Exogenous Ferulic Acid on Selected Individual Phenolic Compounds Stress in Broccoli Microgreens Grown Under Regular Watering and Flooding

**Figure 3:**
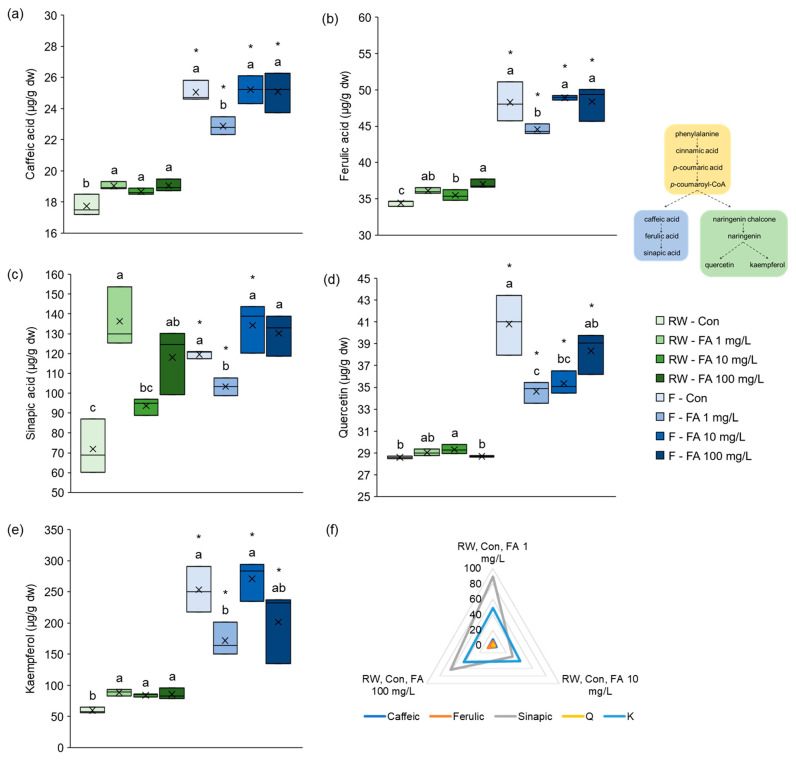
Effects of exogenous ferulic acid on levels of (**a**) caffeic acid, (**b**) ferulic acid, (**c**) sinapic acid, (**d**) quercetin and (**e**) kaempferol in broccoli microgreens grown under regular watering and flooding. (**f**) Percentage of change (%) of individual polyphenolics concentration in broccoli microgreens grown under regular watering conditions after treatment with ferulic acid. Values represent mean ± standard deviation of three biological replicates. Different letters indicate a significant difference among plants grown under the same watering regime (one-way ANOVA, Duncan’s test, *p* ≤ 0.05). An asterisk (*) indicates a significant difference between the same treatment group grown under regular watering regime and flooding (Student’s *t*-test, *p* ≤ 0.05). dw = dry weight; RW = regular watering; Con = control plants irrigated with water; F = flooding; FA = ferulic acid.

The effects of exogenously applied FA on the concentration of individual phenolic compounds, caffeic acid, ferulic acid, sinapic acid, quercetin and kaempferol, under different watering regimes are shown in Figure 3. Regardless of whether treated with FA or not, microgreens grown under flooded conditions had significantly higher concentrations of these individual polyphenolics than those grown under regular watering. The effect of FA was more pronounced in plants watered with regular amounts of water than in plants grown in conditions of flooding. Under regular watering regime, caffeic acid concentration was significantly higher in plants treated with each FA concentration compared to control plants (Figure 3a). On the contrary, under flooding regime, microgreens treated with the lowest concentration of FA (1 mg/L) had significantly lower concentration of caffeic acid compared to all other plants. All plants had notably higher concentration of caffeic acid under flooding than under regular watering regime. Similar to caffeic acid, all treated plants had higher concentration of FA than control plants under regular watering (Figure 3b). However, plants treated with 10 mg/L FA had lower concentration than those treated with 100 mg/L. The levels of FA under flooding behaved the same way as caffeic acid levels—lowest concentration in 1 mg/L FA treated plants, and significantly higher than under regular watering. Flooded control plants synthesized FA as part of the stress response, whereas plants treated with 10 mg/L and 100 mg/L had enough exogenously applied FA so they stopped its synthesis. Those plants could then redirect resources into caffeic and sinapic acid. The concentration of sinapic acid in regularly watered plants was the highest in those treated with 1 mg/L and 100 mg/L FA, and was markedly higher than in control plants (Figure 3c). On the contrary, the concentration was not significantly different between control plants and those treated with 10 mg/L solution. The same pattern observed for caffeic and ferulic acid under flooding was also observed for sinapic acid. However, relations of sinapic acid concentrations under regular watering and flooding were different. The level was significantly higher under flooding than regular watering in control microgreens and those treated with 10 mg/L FA, whereas it was lower in plants treated with 1 mg/L FA. Under regular watering, broccoli microgreens treated with 10 mg/L FA had the highest concentration of quercetin, significantly higher than control and those treated with 100 mg/L (Figure 3d). Under flooding, the opposite was observed. The concentration of quercetin was highest in control plants and those treated with 100 mg/L FA, and lowest in microgreens treated with 1 mg/L. As with caffeic and ferulic acid, the level of quercetin was higher under flooding in all groups compared to regular watering. The pattern for concentration of kaempferol in regularly watered plants was the same as for caffeic acid—higher in plants treated with all concentrations of FA than in control (Figure 3e). Under flooding, the concentration of kaempferol was highest in control plants and those treated with 10 mg/L FA, while it was significantly lower in microgreens treated with 1 mg/L solution. All plants subjected to flooding had more quercetin compared to regularly watered plants. Taken together, under regular watering, sinapic acid and kaempferol were most notably affected by FA treatment of broccoli microgreens (Figure 3f). Application of 1 mg/L and 100 mg/L FA most significantly increased sinapic acid concentration, while application of 10 mg/L FA most significantly increased kaempferol. Since FA is not a direct precursor of kaempferol (Figure 3), we conclude that in the conditions of a flood it functions as a metabolic regulator, and not a substrate for kaempferol increase, through induction of flavonoid biosynthetic pathway. We have previously recorded similar results regarding the effect of flooding in broccoli sprouts grown under flooding conditions for a shorter period (7 days) [14]. The only difference observed was the effect of flooding on sinapic acid concentration: after 7 days of flooding, its concentration decreased, whereas in this study, where the flooding lasted longer (16 days), sinapic acid concentration increased. An increase in ferulic and sinapic acid after flooding was recorded in young Chinese cabbage as well [48]. For comparison, in broccoli irrigated with hot water, FA and quercetin were induced, while sinapic acid and kaempferol were decreased [49]. Based on this, we conclude that FA induction is a general phenylpropanoid stress response, but downstream flavonoid branch allocation is stress-type dependent.

**Figure 4:**
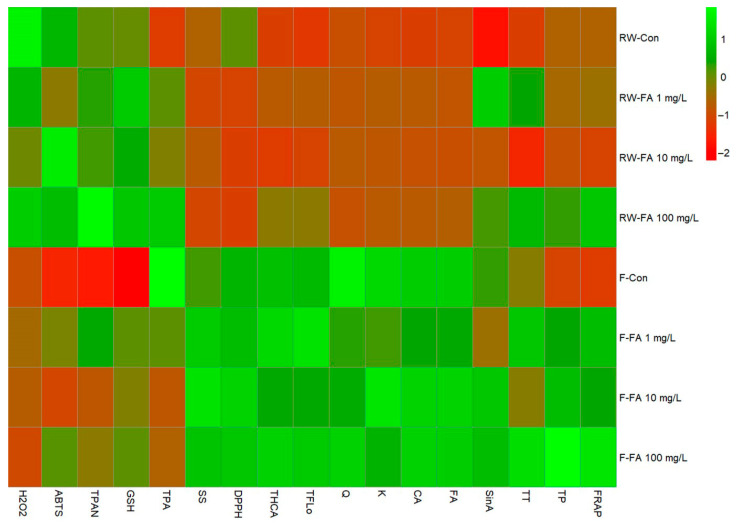
Heatmap showing standardized (z-score) values of broccoli parameters under different treatments. Green indicates relatively higher values and red indicates relatively lower values of the measured variables. TP = total phenolics; TPA = total phenolic acids; THCA = total hydroxycinnamic acids; TFlo = total flavonols; TT = total tannins; TPAN = total proanthocyanidins; SS = soluble sugars; GSH = glutathione; CA = caffeic acid; FA = ferulic acid; SinA = sinapic acid; Q = quercetin; K = kaempferol.

The heatmap of standardized values revealed clear differences between flooding and regular watering treatments in the biochemical responses of broccoli microgreens (Figure 4). Flooded plants generally exhibited higher levels of total hydroxycinnamic acids, flavonols, tannins, soluble sugars, antioxidant capacity measured by DPPH, caffeic acid, ferulic acid, quercetin, kaempferol compared with regularly watered plants. This trend was particularly evident in the F-FA 100 mg/L treatment, which showed consistently positive deviations across most measured variables. In contrast, regularly watered treatments, especially RW-Con and RW-FA 10 mg/L, were characterized by predominantly lower standardized values for phenolic compounds, individual hydroxycinnamic acids (caffeic, ferulic and sinapic acid) and flavonols (quercetin and kaempferol), indicating reduced metabolic activation. A coordinated response was observed among total hydroxycinnamic acids, total flavonols, tannins, caffeic acid, ferulic acid, kaempferol, quercetin and antioxidant capacity measured by DPPH and FRAP, suggesting tight functional linkage between phenylpropanoid metabolism and antioxidant defense mechanisms. The parallel increase in glutathione and phenolic metabolites under high FA supply during flooding suggests enhanced redox buffering capacity and preconditioning of metabolism for antioxidant defenses.

### 2.6. Chemometric Analysis

#### 2.6.1. Interactive Effects of Ferulic Acid and Watering Regime

Two-way factorial ANOVA showed that FA had a significant effect on nine analyzed variables—total phenolics, tannins, proanthocyanidins, antioxidant potential measured by DPPH and FRAP, glutathione, caffeic acid, FA, sinapic acid and quercetin (Table 3). Watering regime had a significant effect on 13 analyzed variables—total phenolics, hydroxycinnamic acids, flavonols, proanthocyanidins, soluble sugars, antioxidant potential measured by DPPH, H_2_O_2_, glutathione, caffeic acid, FA, sinapic acid, quercetin and kaempferol. The interaction between FA and watering regime had a significant effect on 10 analyzed variables—total phenolic acids, proanthocyanidins, soluble sugars, antioxidant potential measured by DPPH, H_2_O_2_, caffeic acid, FA, sinapic acid, quercetin and kaempferol. The response of these parameters to exogenous FA depends on the watering regime. This shows that the efficiency of exogenous FA is strongly dependent on the level of water in soil. It also suggests that watering regime most significantly reprograms broccoli metabolism, while FA served as a selective regulator of phenylpropanoid metabolism and redox balance.

Total phenolic were affected by both FA and watering regime, with FA having a greater impact. On the other hand, total tannins were significantly affected only by FA, while watering regime significantly affected the concentration of total hydroxycinnamic acids and flavonols. The interaction between FA and watering regime had a significant effect on total phenolic acids, whereas both main factors and their interaction significantly influenced the concentration of total proanthocyanidins. Total soluble sugars were affected by both watering regime and the interaction of main factors, with the watering regime having a more pronounced effect. Antioxidant potential measured by different assays was affected by different factors. Antioxidant potential measured by ABTS assay was not significantly affected by any treatment, whereas both main factors and their interaction had a significant effect on antioxidant potential measured by DPPH. The watering regime showed the strongest effect, while FA affected it the least. FA had a significant effect on antioxidant potential measured by FRAP. This indicated that lipophilic antioxidants were more responsive to watering regime and the interaction of watering regime and FA, while FA had a stronger effect on hydrophilic antioxidants [50,51]. Both H_2_O_2_ and GSH were most significantly affected by watering regime, indicating that flooding stress has a strong effect on these markers of oxidative stress. However, GSH was also significantly affected by FA, while the interaction of main factors had a significant effect on H_2_O_2_. Selected individual phenolic compounds were affected by both main factors and their interaction (except for FA effect on kaempferol which was not significant). Watering regime had the most significant effect on caffeic acid, FA, quercetin and kaempferol, whereas the interaction of watering regime and FA had the strongest effect on sinapic acid. These results suggest that both flooding stress and FA treatment affected the concentration of these compounds, with FA effects depending on the watering regime.

#### 2.6.2. Sample Grouping Based on Multivariate Analysis

**Figure 5:**
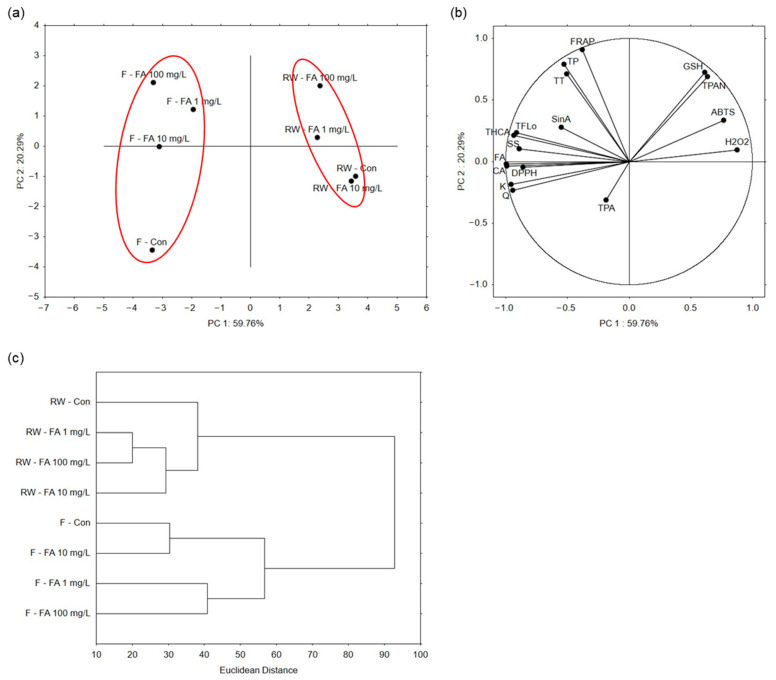
Sample grouping analyses of broccoli microgreens treated with exogenous ferulic acid (FA; 1, 10, 100 mg/L) under regular watering (RW) and flooding (F). Principal component analysis shows (**a**) the sample grouping based on the analyzed parameters, whose grouping is shown in (**b**) part of the figure. (**c**) Hierarchical clustering of samples expressed as Euclidean distance. CA = caffeic acid; FA = ferulic acid; GSH = glutathione; K = kaempferol; Q = quercetin; SinA = sinapic acid; SS = soluble sugars; TFlo = total flavonols; THCA = total hydroxycinnamic acids; TP = total phenolics; TPA = total phenolic acids; TPAN = total proanthocyanidins; TT = total tannins.

Principal component analysis (PCA) was performed to reduce dimensionality and explore the overall variation among samples. The first two principal components explained 80.05% of the total variance (PC 1: 59.76%, PC 2: 20.29%) (Figure 5a). Broccoli microgreens grown under regular watering formed one cluster, while those grown under flooding conditions formed a separate cluster, indicating that microgreens shared similar profiles within each watering regime. Within the regular watering cluster, control plants and those treated with 10 mg/L FA clustered closely, whereas plants treated with the highest concentration (100 mg/L) were the most separated. In the flooding cluster, microgreens treated with each FA concentration grouped together, while control plants were separated. As in the regular watering cluster, plants treated with the highest FA concentration were furthest from control, indicating the most pronounced differences. PC 1 primarily drove the separation between the regular watering and flooding clusters, whereas PC 2 mainly contributed to the separation between control and treated plants. The level of H_2_O_2_ contributed the most to the separation of regularly watered microgreens treated with 1 mg/L FA, whereas glutathione and total proanthocyanidins of those treated with 100 mg/L FA (Figure 5b). Variables that contributed the most to the separation of flooded microgreens treated with 10 mg/L FA were concentration of ferulic and caffeic acid, and antioxidant potential measured by DPPH. Concentration of sinapic acid contributed the most to the separation of flooded plants treated with 1 mg/L FA.

Hierarchical clustering (HC) was applied to reveal which samples were most similar based on all the measured parameters (Figure 5c). Consistent with PCA, it showed a clear separation of microgreens grown under regular watering and those grown under flooding conditions. Under regular watering, plants treated with FA formed a cluster separate from control plants. Microgreens treated with 1 mg/L and 100 mg/L FA solutions were the most similar (Euclidean distance 20). Under flooding, control plants and those treated with 10 mg/L FA formed one, and plants treated with 1 mg/L and 100 mg/L FA another cluster. Control plants and those treated with 10 mg/L FA were the most similar (Euclidean distance 30). Microgreens grown under regular watering were more similar to each other (Euclidean distance 38) than those grown under flooding (Euclidean distance 57).

#### 2.6.3. Correlation Patterns Among Measured Parameters

**Figure 6:**
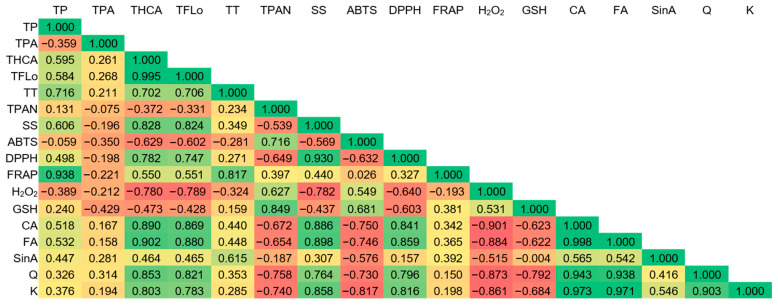
Pearson’s correlation coefficients between measured variables in broccoli microgreens treated with exogenous ferulic acid grown under regular watering and flooding. TP = total phenolics; TPA = total phenolic acids; THCA = total hydroxycinnamic acids; TFlo = total flavonols; TT = total tannins; TPAN = total proanthocyanidins; SS = soluble sugars; GSH = glutathione; CA = caffeic acid; FA = ferulic acid; SinA = sinapic acid; Q = quercetin; K = kaempferol.

Pearson’s correlation coefficients between measured parameters are shown in Figure 6. A very strong positive correlation observed between total hydroxycinnamic and ferulic and caffeic acid was expected since both ferulic and caffeic acid belong to hydroxycinnamic acids. Similarly, there was a very strong positive correlation between total flavonols and quercetin. Even though sinapic acid is a hydroxycinnamic acid, the correlation coefficient was a lot lower and this phytochemical behaved much differently than total hydroxycinnamic acids and other measured individual compounds. Under flooding, a significant accumulation of phenolic compounds (total hydroxycinnamic acids and flavonols, as well as caffeic acid, ferulic acid, quercetin and kaempferol) and soluble sugars was observed in response to stress. Thus, all of these parameters showed a very strong positive correlation. Phenolic compounds act as antioxidants and mentioned parameters were very strongly positively correlated with antioxidant potential measured by DPPH as well. On the other hand, total phenolics and total tannins were very strongly correlated with antioxidant potential measured by FRAP and showed a distinct pattern. A very strong positive correlation was observed between total proanthocyanidins and glutathione. The level of H_2_O_2_ was very strongly negatively correlated with the concentration of caffeic acid, ferulic acid, quercetin and kaempferol. While the level of those bioactive compounds was much higher under flooding that in regular watering, the opposite was observed for H_2_O_2_.

## 3. Materials and Methods

### 3.1. Plant Material and Growth Conditions

*Brassica oleracea* L. convar. *botrytis* (L.) Alef. var. *cymosa* Duch. seeds Art. No. 424430 were purchased from International Seeds Processing GmbH (Quedlinburg, Germany). Seeds were sown in pots containing sterile soil substrate Stender B400 and grown in a greenhouse at 22–25 °C and 65% humidity, under long day conditions (16 h day/8 h night). Seven days after sowing, exogenous application of ferulic acid and different watering regimes started. Plants were irrigated with three concentrations of ferulic acid (1, 10 and 100 mg/L), while control plants were irrigated with water. For different watering regime plants exposed to flooding pots were constantly immersed in water or ferulic acid solution, while control plants were normally irrigated. All plants were irrigated with equal volumes of water or ferulic acid solution throughout the treatment period. Treatments lasted for 16 days, after which the aerial parts of plants were sampled, frozen in liquid nitrogen and lyophilized using an Alpha 1–2 LSC basic freeze-dryer (Martin Christ Gefriertrocknungsanlagen GmbH, Osterode am Harz, Germany). Lyophilized broccoli samples were powdered and extracts were prepared for different analysis methods. Cultivation was conducted from March to May 2025.

### 3.2. Quantification of Polyphenolic Compounds

Polyphenolics were quantified spectrophotometrically using a Fluostar Optima microplate reader (BMG LABTECH) in 70% ethanolic extracts (30 mg/mL). Results were expressed as equivalents of corresponding standard per gram of dry weight (mg/g dw).

Total phenolics were determined by the Folin–Ciocâlteu method [52,53] at 740 nm, using gallic acid (GA) as a standard (0.02–1.28 mg/mL) and the results were expressed as mg GAE/g dw.

Total phenolic acids were determined by the Arnow method [54] at 485 nm, using caffeic acid (CA) as a standard (0.04–2.56 mg/mL) and the results were expressed as mg CAE/g dw.

For determination of total hydroxycinnamic acids and flavonols extracts at a concentration of 6 mg/mL were used, according to Howard et al. [55]. Hydroxycinnamic acids were quantified at 320 nm using cinnamic acid (CinA) as standard (0.02–1.28 mg/mL). Flavonols were quantified at 355 nm using quercetin (Q) as a standard (0.005–0.32 mg/mL). Results were expressed as mg CinAE/mg dw and mg QE/g dw.

Total tannins were quantified according to Galvão et al. [56] at 740 nm using GA standard (0.01–0.64 mg/mL) and expressed as mg GAE/g dw.

Proanthocyanidins were quantified following Weidner et al. [57] at 485 nm, using catechin (Cat) standard (0.01–0.64 mg/mL) and expressed as mg CatE/g fw.

### 3.3. Quantification of Total Soluble Sugars

Soluble sugars content was determined in 70% ethanolic extracts (6 mg/mL) following Dubois et al. [58] at 485 nm, using sucrose (Suc) as a standard (0.02–1.28 mg/mL) and results were expressed as mg Suc/g dw.

### 3.4. Determination of Antioxidant Potential

Antioxidant potential was assessed spectrophotometrically using a Fluostar Optima microplate reader in 70% ethanolic extracts using ABTS [59], DPPH [60] and FRAP [61] assays, as described in Šola et al. [10]. For the ABTS and DPPH assay extracts a concentration of 30 mg/mL were used and results were expressed as percentage of inhibition. For FRAP assay extracts a concentration of 6 mg/mL were used and results were expressed as percentage of reduction. Results were calculated according to the following equations:

(1) $$\%\;inhibition=\frac{{Abs}_{0}-{Abs}_{t}}{{Abs}_{0}}\times 100$$

(2) $$\%\;reduction=\frac{{Abs}_{t}-{Abs}_{0}}{{Abs}_{t}}\times 100$$

where Abs_t_ = absorbance of the extract and Abs_0_ = absorbance of 70% ethanol.

### 3.5. Quantification of Hydogen Peroxide and Glutathione Level

Hydrogen peroxide and glutathione were quantified spectrophotometrically using a Fluostar Optima microplate reader in 70% ethanolic extracts (30 mg/mL).

H_2_O_2_ level was determined according to Junglee et al. [62] at 405 nm, using H_2_O_2_ standard (0.12–5 mM) and results were expressed as mM/g dw.

Glutathione concentration was determined according to Malar et al. [63] using glutathione as a standard (0.01–1 mg/mL) and results were expressed as mg/g dw.

### 3.6. Reversed-Phase High Performance Liquid Chromatography Analysis

Before reversed-phase high performance liquid chromatography (RP-HPLC) analysis, samples were hydrolyzed using 1.2 M HCl for 2 h at 80 °C and 300 rpm. The solutions were centrifuged and supernatants were taken for analysis.

RP-HPLC analysis was performed on an Agilent 1260 Infinity II HPLC with vialsampler G7129A, quaternary pump VL G7111A with an operating pressure of up to 600 bar, multicolumn thermostat and diode array detector G7115A (Agilent Technologies, Santa Clara, California, USA). The software used was OpenLab CDS LC Chemstation Upgrade, version C02.05. The components were separated using guard column Zorbax Rx-C18 (4.6 mm × 12.5 mm, d(particle) = 5 μm) and a non-polar column Poroshell 120 SB C-18 (4.6 mm × 75 mm, d(particle) = 2.7 μm). The solvents used were 0.2% acetic acid (A) and 80% methanol mixed with 0.2% acetic acid (B), purchased from Sigma-Aldrich GmbH, Merck Taufkirchen, Germany). The solvent gradient was the same as in our previous study [10]. A volume of 25 μL of each extract was injected at a flow rate of 1.0 mL/min. The column temperature was set to 30 °C and the absorbance was measured at λ = 310 nm for caffeic, ferulic and sinapic acid, and λ = 360 for quercetin and kaempferol. The components were identified based on their retention times, UV spectra compared to commercial standards, and co-injections with standards. For quantification purposes, seven known concentrations of the mixed standard solution were made and calibration curves were calculated. The calibration curves are shown in Table S1 along with their *R*^2^ values. Results are expressed in μg/g of plant dry mass.

### 3.7. Statistical Analysis

Data were obtained from four biological replicates, each analyzed with three technical replicates, except for HPLC analyses which were analyzed in three biological replicates. All results are presented as absolute values, without calculation of net changes, with statistical comparisons to controls and between regimes. Statistical analyses were performed using Statistica software (version 14.1.0.8; TIBCO Software Inc., Palo Alto, CA, USA). To evaluate the effect of watering regime, Student’s *t*-test was applied. The effect of exogenous ferulic acid in plants grown under the same watering regime (either regular watering or flooding) was assessed using one-way ANOVA followed by Duncan’s multiple range test (DMRT). To determine the relative contribution of ferulic acid and watering regime to the variation in the analyzed parameters, as well as their potential interaction, a two-way factorial ANOVA was conducted. Differences were considered statistically significant at the *p* ≤ 0.05 level. Principal component analysis (PCA) and hierarchical clustering (HC) using Euclidean distance were performed to assess sample similarity and grouping. Pearson’s correlation coefficients were calculated to evaluate relationships between parameters, with values of 0.80–1.00 considered very high [64].

## 4. Conclusions

Exogenous FA exhibits biostimulant-like effects in broccoli microgreens, particularly under flooding stress. FA enhanced antioxidant capacity and redox homeostasis by promoting glutathione accumulation and stimulating phenylpropanoid metabolism. Although flooding is generally associated with increased ROS production, H_2_O_2_ levels did not show consistent elevation in FA-treated plants, whereas glutathione and phenolic antioxidants were markedly enhanced. This pattern suggests that FA improves redox homeostasis by strengthening antioxidant buffering systems, thereby limiting excessive ROS accumulation under hypoxic stress conditions. The effects of FA were concentration-dependent and strongly influenced by soil water status, with different responses observed in regularly watered versus flooded plants. These findings highlight FA potential to mitigate flooding-induced oxidative stress and support its use as a strategy to enhance crop resilience under climate change.

## Figures and Tables

**Table 1:** Effect of exogenous ferulic acid on different groups of phenolic compounds in broccoli microgreens grown under regular watering and flooding.

Total phenolics (mg GAE/g dw)			
RW-Con	12.17 ± 0.64 a	F-Con	11.49 ± 0.75 c
RW-FA 1 mg/L	12.34 ± 1.42 a	F-FA 1 mg/L	13.94 ± 0.95 b
RW-FA 10 mg/L	11.79 ± 1.35 a	F-FA 10 mg/L	14.52 ± 1.53 ab *
RW-FA 100 mg/L	13.76 ± 1.54 a	F-FA 100 mg/L	16.29 ± 1.95 a
Total phenolic acids (mg CAE/g dw)			
RW-Con	7.76 ± 0.63 b	F-Con	10.73 ± 3.17 a
RW-FA 1 mg/L	9.03 ± 0.91 a	F-FA 1 mg/L	9.03 ± 0.76 a
RW-FA 10 mg/L	8.77 ± 0.88 ab	F-FA 10 mg/L	8.15 ± 0.31 a
RW-FA 100 mg/L	9.91 ± 0.26 a	F-FA 100 mg/L	8.34 ± 0.39 a *
Total hydroxycinnamic acids (mg CinAE/g dw)			
RW-Con	77.66 ± 10.60 a	F-Con	96.05 ± 10.83 a
RW-FA 1 mg/L	81.21 ± 2.22 a	F-FA 1 mg/L	99.94 ± 13.95 a *
RW-FA 10 mg/L	76.89 ± 3.16 a	F-FA 10 mg/L	92.56 ± 7.21 a *
RW-FA 100 mg/L	85.65 ± 8.34 a	F-FA 100 mg/L	98.61 ± 10.39 a
Total flavonols (mg QE/g dw)			
RW-Con	8.64 ± 1.74 a	F-Con	11.44 ± 1.20 a *
RW-FA 1 mg/L	9.42 ± 0.74 a	F-FA 1 mg/L	12.36 ± 1.85 a *
RW-FA 10 mg/L	8.87 ± 0.70 a	F-FA 10 mg/L	11.03 ± 0.99 a *
RW-FA 100 mg/L	10.01 ± 1.73 a	F-FA 100 mg/L	11.81 ± 1.22 a
Total tannins (mg GAE/g dw)			
RW-Con	6.34 ± 0.81 a	F-Con	6.88 ± 0.34 a
RW-FA 1 mg/L	7.26 ± 0.91 a	F-FA 1 mg/L	7.58 ± 0.61 a
RW-FA 10 mg/L	6.16 ± 1.19 a	F-FA 10 mg/L	6.89 ± 0.42 a
RW-FA 100 mg/L	7.46 ± 1.09 a	F-FA 100 mg/L	7.80 ± 0.90 a
Total proanthocyanidins (mg CatE/g dw)			
RW-Con	1.67 ± 0.17 b	F-Con	1.26 ± 0.08 d *
RW-FA 1 mg/L	1.73 ± 0.12 b	F-FA 1 mg/L	1.76 ± 0.07 a
RW-FA 10 mg/L	1.71 ± 0.08 b	F-FA 10 mg/L	1.45 ± 0.07 c *
RW-FA 100 mg/L	2.07 ± 0.10 a	F-FA 100 mg/L	1.59 ± 0.06 b *

Values represent average ± standard deviation of four biological replicates. Different letters indicate a significant difference among plants grown under the same watering regime (one-way ANOVA, Duncan’s test, *p* ≤ 0.05). An asterisk (*) indicates a significant difference between the same treatment group grown under regular watering regime and flooding (Student’s *t*-test, *p* ≤ 0.05). GAE = gallic acid equivalent; dw = dry weight; RW = regular watering; Con = control plants irrigated with water; F = flooding; FA = ferulic acid; CAE = caffeic acid equivalent; CinAE = cinnamic acid equivalent; QE = quercetin equivalent; CatE = catechin equivalent.

**Table 2:** Effect of exogenous ferulic acid on antioxidant capacity of broccoli microgreens grown under regular watering and flooding.

ABTS (% inhibition)			
RW-Con	32.24 ± 3.86 a	F-Con	27.47 ± 1.93 a
RW-FA 1 mg/L	30.13 ± 5.67 a	F-FA 1 mg/L	30.47 ± 1.58 a
RW-FA 10 mg/L	34.03 ± 2.94 a	F-FA 10 mg/L	28.47 ± 4.08 a
RW-FA 100 mg/L	32.38 ± 8.74 a	F-FA 100 mg/L	31.03 ± 7.31 a
DPPH (% inhibition)			
RW-Con	57.40 ± 2.44 a	F-Con	60.69 ± 2.53 a
RW-FA 1 mg/L	50.42 ± 2.07 b	F-FA 1 mg/L	61.44 ± 2.06 a *
RW-FA 10 mg/L	50.03 ± 1.81 b	F-FA 10 mg/L	63.56 ± 3.25 a *
RW-FA 100 mg/L	50.22 ± 2.26 b	F-FA 100 mg/L	62.44 ± 0.56 a *
FRAP (% reduction)			
RW-Con	61.01 ± 2.38 a	F-Con	59.63 ± 4.45 b
RW-FA 1 mg/L	61.58 ± 3.04 a	F-FA 1 mg/L	64.50 ± 0.84 a
RW-FA 10 mg/L	59.92 ± 4.79 a	F-FA 10 mg/L	63.67 ± 2.21 ab
RW-FA 100 mg/L	64.89 ± 1.98 a	F-FA 100 mg/L	66.17 ± 2.27 a

Values represent average ± standard deviation of four biological replicates. Different letters indicate a significant difference among plants grown under the same watering regime (one-way ANOVA, Duncan’s test, *p* ≤ 0.05). An asterisk (*) indicates a significant difference between the same treatment group grown under regular watering regime and flooding (Student’s *t*-test, *p* ≤ 0.05). RW = regular watering; Con = control plants irrigated with water; F = flooding; FA = ferulic acid.

**Table 3:** Two-way ANOVA showing the effect of the main factors (exogenous ferulic acid and watering regime) and their interaction on different groups of phytochemicals and antioxidant activity of broccoli microgreens.

Parameter	Source of Variation	SS	F	*p*
Total phenolics	ferulic acid	41.375 ***	7.751 ***	0.000865 ***
	watering regime	19.071 **	10.719 **	0.003210 **
	FA × watering	14.673	2.749	0.064905
Total phenolic acids	ferulic acid	2.942	0.606	0.617394
	watering regime	0.304	0.188	0.668308
	FA × watering	23.087 **	4.758 **	0.009653 **
Total hydroxycinnamic acids	ferulic acid	275.3	1.097	0.369401
	watering regime	2161.8 ***	25.857 ***	0.000034 ***
	FA × watering	43.5	0.174	0.913257
Total flavonols	ferulic acid	6.586	1.222	0.323135
	watering regime	47.023 ***	26.183 ***	0.000031 ***
	FA × watering	1.717	0.319	0.811734
Total tannins	ferulic acid	7.565 *	3.631 *	0.027239 *
	watering regime	1.889	2.720	0.112107
	FA × watering	0.223	0.107	0.955252
Total proanthocyanidins	ferulic acid	0.64212 ***	21.963 ***	0.000000 ***
	watering regime	0.62651 ***	64.288 ***	0.000000 ***
	FA × watering	0.31153 ***	10.656 ***	0.000121 ***
Total soluble sugars	ferulic acid	58.55	2.448	0.088234
	watering regime	1275.83 ***	160.060 ***	0.000000 ***
	FA × watering	105.74 *	4.422 *	0.013044 *
ABTS	ferulic acid	17.34	0.222	0.880282
	watering regime	64.21	2.464	0.129556
	FA × watering	46.91	0.600	0.621174
DPPH	ferulic acid	46.6 *	3.09 *	0.045975 *
	watering regime	802.1 ***	159.75 ***	0.000000 ***
	FA × watering	127.1 ***	8.44 ***	0.000527 ***
FRAP	ferulic acid	116.7 *	4.30 *	0.014576 *
	watering regime	21.6	2.39	0.135318
	FA × watering	30.7	1.13	0.355869
Hydrogen peroxide	ferulic acid	232.3	2.154	0.119796
	watering regime	2255.2 ***	62.726 ***	0.000000 ***
	FA × watering	491.3 *	4.555 *	0.011563 *
Glutathione	ferulic acid	886.98 **	6.3654 **	0.002497 **
	watering regime	670.98 ***	14.4460 ***	0.000870 ***
	FA × watering	181.71	1.3040	0.296049
Caffeic acid	ferulic acid	4.76 *	3.25 *	0.049550 *
	watering regime	211.06 ***	431.81 ***	0.000000 ***
	FA × watering	10.09 **	6.88 **	0.003443 **
Ferulic acid	ferulic acid	19.38 *	3.56 *	0.038002 *
	watering regime	829.62 ***	457.53 ***	0.000000 ***
	FA × watering	27.70 *	5.09 *	0.011558 *
Sinapic acid	ferulic acid	2789.3 **	7.482 **	0.002383 **
	watering regime	1706.9 **	13.736 **	0.001917 **
	FA × watering	6021.3 ***	16.152 ***	0.000042 ***
Quercetin	ferulic acid	29.50 **	5.84 **	0.006826 **
	watering regime	419.80 ***	249.30 ***	0.000000 ***
	FA × watering	44.43 **	8.80 **	0.001122 **
Kaempferol	ferulic acid	7274.0	2.9717	0.063100
	watering regime	125,807.8 ***	154.1893 ***	0.000000 ***
	FA × watering	13,065.9 **	5.3378 **	0.009680 **

Asterisk indicates significance levels: * *p* ≤ 0.05, ** *p* ≤ 0.01, *** *p* ≤ 0.001.

## Data Availability

The data that support the findings of this study are available from the corresponding author I.Š., upon request.

## Supplementary Materials

The following supporting information can be downloaded at: https://www.mdpi.com/article/10.3390/plants15091323/s1, Table S1: Calibration curves and *R*^2^ values of polyphenolic standard compounds.

## Abbreviations

The following abbreviations are used in this manuscript:

ROS	Reactive oxygen species
FA	Ferulic acid
PCA	Principal component analysis
HC	Hierarchical clustering
GAE	Gallic acid equivalent
dw	Dry weight
RW	Regular watering
Con	Control plants irrigated with water
F	Flooding
CAE	Caffeic acid equivalents
CinAE	Cinnamic acid equivalent
QE	Quercetin equivalent
CatE	Catechin equivalent
SucE	Sucrose equivalent
ABTS	2,2′-azino-bis(3-ethylbenzothiazoline-6-sulfonic acid)
DPPH	2,2-diphenyl-1-picrylhydrazyl
FRAP	Ferric reducing antioxidant power
CA	Caffeic acid
GSH	Glutathione
K	Kaempferol
Q	Quercetin
SinA	Sinapic acid
SS	Soluble sugars
TFlo	Total flavonols
THCA	Total hydroxycinnamic acids
TP	Total phenolics
TPA	Total phenolic acids
TPAN	Total proanthocyanidins
TT	Total tannins
