# Supplementary material for: Exogenous Ferulic Acid Mitigates Flooding Stress in Broccoli via GSH-Mediated Redox Homeostasis
Source: Plants (Basel). 2026 Apr 25;15(9):1323. doi: 10.3390/plants15091323 (PMC13165301; doi:10.3390/plants15091323)
Supplement: Supplementary file 1 [file plants-15-01323-s001.zip › plants-4236774-supplementary.pdf]

Table S1. Calibration curves and R^2 values of polyphenolic standard compounds.

Component	Calibration curve	R^2
Caffeic acid	y = 13851x – 149.78	0.9995
Ferulic acid	y = 16304x – 339.23	0.9993
Sinapic acid	y = 17414x - 14.767	0.9989
Quercetin	y = 6241.5x – 114.62	0.995
Kaempferol	y = 5851.5x - 173.58	0.998
